# The Role of Translocator Protein TSPO in Hallmarks of Glioblastoma

**DOI:** 10.3390/cancers12102973

**Published:** 2020-10-14

**Authors:** Laura-Marie Ammer, Arabel Vollmann-Zwerenz, Viktoria Ruf, Christian H. Wetzel, Markus J. Riemenschneider, Nathalie L. Albert, Philipp Beckhove, Peter Hau

**Affiliations:** 1 Wilhelm Sander-NeuroOncology Unit and Department of Neurology, University Hospital Regensburg, 93053 Regensburg, Germany; laura-marie.ammer@ukr.de (L.-M.A.); arabel.vollmann@ukr.de (A.V.-Z.); 2 Center for Neuropathology and Prion Research, Ludwig Maximilians University of Munich, 81377 Munich, Germany; viktoria.ruf@med.uni-muenchen.de; 3 Molecular Neurosciences, Department of Psychiatry and Psychotherapy, University of Regensburg, 93053 Regensburg, Germany; christian.wetzel@ukr.de; 4 Department of Neuropathology, Regensburg University Hospital, 93053 Regensburg, Germany; markus.riemenschneider@ukr.de; 5 Department of Nuclear Medicine, Ludwig-Maximilians-University Munich, 81377 Munich, Germany; nathalie.albert@med.uni-muenchen.de; 6 Regensburg Center for Interventional Immunology (RCI) and Department Internal Medicine III, University Hospital Regensburg, 93053 Regensburg, Germany; philipp.beckhove@ukr.de

**Keywords:** TSPO, glioblastoma, hallmarks of cancer, diagnostic marker

## Abstract

**Simple Summary:**

The translocator protein (TSPO) has been under extensive investigation as a specific marker in positron emission tomography (PET) to visualize brain lesions following injury or disease. In recent years, TSPO is increasingly appreciated as a potential novel therapeutic target in cancer. In Glioblastoma (GBM), the most malignant primary brain tumor, TSPO expression levels are strongly elevated and scientific evidence accumulates, hinting at a pivotal role of TSPO in tumorigenesis and glioma progression. The aim of this review is to summarize the current literature on TSPO with respect to its role both in diagnostics and especially with regard to the critical hallmarks of cancer postulated by Hanahan and Weinberg. Overall, our review contributes to a better understanding of the functional significance of TSPO in Glioblastoma and draws attention to TSPO as a potential modulator of treatment response and thus an important factor that may influence the clinical outcome of GBM.

**Abstract:**

Glioblastoma (GBM) is the most fatal primary brain cancer in adults. Despite extensive treatment, tumors inevitably recur, leading to an average survival time shorter than 1.5 years. The 18 kDa translocator protein (TSPO) is abundantly expressed throughout the body including the central nervous system. The expression of TSPO increases in states of inflammation and brain injury due to microglia activation. Not least due to its location in the outer mitochondrial membrane, TSPO has been implicated with a broad spectrum of functions. These include the regulation of proliferation, apoptosis, migration, as well as mitochondrial functions such as mitochondrial respiration and oxidative stress regulation. TSPO is frequently overexpressed in GBM. Its expression level has been positively correlated to WHO grade, glioma cell proliferation, and poor prognosis of patients. Several lines of evidence indicate that TSPO plays a functional part in glioma hallmark features such as resistance to apoptosis, invasiveness, and proliferation. This review provides a critical overview of how TSPO could regulate several aspects of tumorigenesis in GBM, particularly in the context of the hallmarks of cancer proposed by Hanahan and Weinberg in 2011.

## 1. Introduction

Glioblastoma (GBM) is the most common and most aggressive primary brain cancer with a very low life expectancy after diagnosis [1]. It can arise as primary or as secondary tumors through the progression from a lower grade glial tumor [2] and is characterized by highly infiltrative and invasive growth, necrosis, and microvascular proliferation [3,4,5,6]. GBM’s complex biology makes it significantly challenging to treat, and despite multimodal treatment, only a little progress has been made towards better prognoses. The median survival time is 14–16 months with a 5-year overall survival of 9.8% [7,8].

GBM´s intrinsic heterogeneity [9,10] reflects in a plethora of distinct genetic events and pathways that are context-specific (reviewed in [11]). For instance, mutations in the isocitrate dehydrogenase 1 or 2 gene (*IDH1*, *2*) impair metabolism, leading to a decreased production of bioenergy and intermediates (reviewed in [12,13]) and a better outcome for patients [14,15].

It is becoming increasingly evident that mitochondria are involved in the vast majority of the pathogenic events of GBM. Mitochondria bear key biosynthetic and metabolic functions and are central regulators of cell death, inflammation, immunity, and migration (reviewed in [16]). Therefore, mitochondrial dysfunctions can not only be considered pathogenic but could also constitute potential therapeutic targets.

Due to its location in the outer mitochondrial membrane (OMM), the role of translocator protein (TSPO) has been extensively studied within the last two decades. It has thereby been implicated with a broad spectrum of functions such as steroid synthesis [17], regulation of proliferation [18,19], apoptosis [20,21,22] and migration [23], as well as mitochondrial functions such as mitochondrial respiration [24,25] and oxidative stress regulation [26].

The functional relevance of TSPO is also reflected in its distinct expression levels within human diseases, including neurodegenerative disorders [27] and malignant gliomas [28], where TSPO expression levels positively correlate with the malignancy of tumors [19,29].

This article hypothesizes that TSPO in GBM relates to many of the hallmarks of cancer, published first by Hanahan and Weinberg in a groundbreaking, conceptual article in 2000 [30]. Here, the highly diverse and complex nature of cancer was reduced to a number of underlying, essential principles. In 2011, the authors added two biological capabilities, one of them labeled as a reprogramming of cellular energetics [31], which might have specific relevance in the context of TSPO.

**Figure 1:**
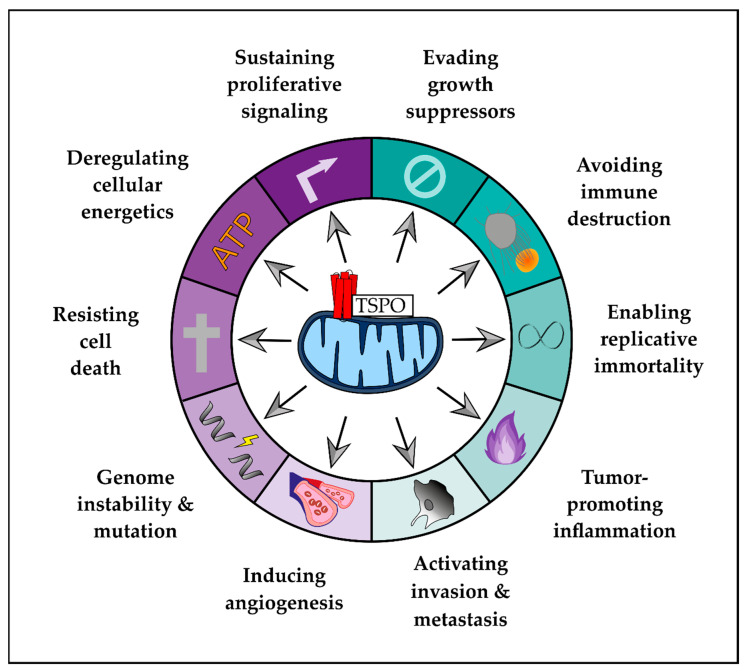
Translocator protein (TSPO) and the hallmarks of cancer. This illustration summarizes the ten hallmarks of cancer as proposed by Hanahan and Weinberg [31], which might be modulated by the translocator protein TSPO (adapted from [31]).

This review provides an overview of how TSPO could be involved in the modulation of malignant hallmarks of GBM, also shedding light on TSPO as a hallmark-related diagnostic marker (Figure 1).

### 1.1. Glioblastoma Pathophysiology

Over the last years, great progress has been made in understanding the pathology of GBM. As described by Hanahan and Weinberg, the malignant transformation from a normal cell into a tumor, in this case, GBM, results from the sequential accumulation of molecular aberrations [30]. Around 90% of GBM cases develop de novo (primary GBM) by multistep tumorigenesis [32,33]. These primary GBM are characterized by epidermal growth factor receptor (EGFR) amplifications, inactivation of phosphate and tensin homolog (PTEN), and loss of heterozygosity (LOH) on chromosome 10q [34,35]. A subset of ~10% of GBM develops as a secondary neoplasm through the progression from low glial grade tumors (WHO grade II) or anaplastic glial tumors (WHO grade III) [33,34]. Secondary GBM exhibit platelet-derived growth factor receptor (PDGFR) activation and p53 mutations, which are rare in de novo GBM [32,36,37]. In addition, *IDH* mutations appear frequently in secondary GBM, contributing to the slightly better outcome of secondary in comparison to primary tumors [38,39].

Despite these differences, most of the genetic alterations in primary and secondary GBM can be assigned to a common set of functional pathways that regulate cellular proliferation and survival, as well as invasion and angiogenesis. These aberrations include the activation of receptor tyrosine kinase (RTK) genes and phosphoinositol-3-kinase (PI3K) pathways, the inactivation of the p53 pathway, and the inactivation of the retinoblastoma (RB) suppressor pathway [40]. The enrichment of alterations in these pathways can be linked to distinct molecular subtypes of GBM, namely proneural, classical, and mesenchymal. The proneural subtype, for instance, mainly carries *PDGFRA*/*IDH*/*TP53* mutations, and *EGFR* and *NF1/TP53* mutations were represented in the classical and mesenchymal subtypes, respectively [41]. Out of the three subtypes, patients diagnosed with the proneural subtype have a better outcome, while the mesenchymal subtype leads to the most devastating prognosis [41,42].

Further important deregulated pathways in GBM include the signal transducer and activator of transcription 3 (STAT3), which is upregulated in GBM [43]. STAT3 signaling can be stimulated by many growth factors and cytokines and leads to the activation of multiple genes associated with cell cycle, anti-apoptosis, cell survival, angiogenesis, migration, and invasion (reviewed in [44]). Another crucial transcription factor is nuclear factor-κB (NF-κB), which regulates a broad range of genes linked to proliferation, inflammation, differentiation, motility, and survival (reviewed in [45]). In GBM, NF-κB is aberrantly activated and has been implicated with the maintenance of cancer stem cells, stimulation of invasion, promotion of mesenchymal identity, and resistance to therapy [46,47,48,49,50]. Both STAT3 and NF-κB, have been connected to the mesenchymal GBM subtype [51].

Changes in critical signaling pathways, as well as characteristic mutations identified in each subtype, have a great impact on the hallmarks of GBM. They enable the tumor to context-dependent uncontrolled cellular proliferation, diffuse infiltration, a tendency for necrosis, robust angiogenesis, resistance to apoptosis, and genomic instability [52].

### 1.2. Translocator Protein TSPO 

The 18 kDa translocator protein TSPO is an evolutionary well-conserved protein which comprises 169 amino acids and is organized in five tightly packed α-helical transmembrane domains [53]. It can be found in monomer, dimer, and polymer states [54]. TSPO is ubiquitously expressed and particularly abundant in steroid synthesizing tissues and cells such as gonads and adrenal cells, whereas in the central nervous system (CNS), it is mainly expressed in microglial cells [55].

It was first recognized for its role in cholesterol transport [56]. Cholesterol [57], porphyrins [58], and the diazepam binding inhibitor (DBI) [59] are endogenous TSPO ligands, and TSPO also has a high affinity for a wide range of synthetic ligands such as PK11195 or Ro5-4864, which were primarily developed as neuroimaging agents [17], and etifoxine (Stresam), which was approved as an anxiolytic and anti-depressant for anxiety disorders in some countries [60].

The expression of TSPO is regulated on multiple levels. The GC-rich promotor contains binding sites for several transcription factors including Sp (Specificity protein) 1, Sp3, and Sp4 [61]. There is strong evidence that mainly the PKCε-ERK1/-AP1/STAT3 signaling pathway affects TSPO transcription by upregulation of Ets and Sp1/Sp3 transcription factors [62,63]. In addition, TSPO gene amplification has been shown in human breast cancer cell lines [64] and metastases [65]. Finally, epigenetic regulation of TSPO expression via aberrant promotor methylation or histone modifications has been proposed, since the histone deacetylase inhibitor, TSA, induced TSPO promotor activity in human breast cancer cell lines [61], However, this has not been replicated in GBM so far.

Several studies have found a strong correlation between TSPO expression levels and aggressive cancer phenotypes. Indeed, TSPO is frequently upregulated in gliomas with the highest expression in GBM [28] and the level of TSPO expression correlates with proliferative and apoptotic indices and prognosis [19,29,66]. Noteworthy, TSPO expression levels also differ significantly between molecular subtypes of GBM with the highest expression in the mesenchymal subtype [66].

## 2. Role of TSPO in Hallmarks of GBM

### 2.1. Enabling Characteristics 

#### 2.1.1. Genome Instability and Mutation

Accumulation of genetic mutations and chromosome rearrangements play a critical role in the initiation and progression of cancer by severely contributing to genomic instability. Deficiency in DNA repair and recombination pathways as well as in cell cycle checkpoints and apoptosis lead to a high rate of genomic instability in cancer cells [67]. In addition, reactive oxygen species (ROS) can contribute to genomic instability by causing DNA damage via base and sugar modifications resulting in DNA strand breaks (reviewed in [68,69]).

Tumors, including GBM, produce elevated levels of ROS in comparison to non-cancerous cells [70,71]. Mitochondria are one of the major sources of endogenous ROS in cancer [72,73]. Already early on, TSPO has been linked to oxidative stress and ROS production [74]. In U118MG human glioblastoma cells, TSPO knockdown attenuated the ROS generation of cobalt chloride (CoCl_2_) [21]. Overexpression of TSPO, on the other hand, increased ROS production [75]. Notably, *TSPO* transcription itself is regulated by transcription factors that operate downstream of ROS-sensitive pathways such as protein kinase Cε (PKCε) [63,76], suggesting a feedback loop between TSPO expression and ROS signaling [77].

A number of studies indicate that TSPO is linked to oxidative stress homeostasis regulation. However, to what extent this contributes to the genomic instability and mutational burden of GBM is not yet known. Of note, other mechanisms that further contribute to genomic instability, e.g., the deregulation of apoptosis and cell cycle, are also likely to be modulated by TSPO and will be discussed elsewhere in this review.

#### 2.1.2. Tumor Promoting Inflammation

GBM is highly infiltrated by microglia and macrophages, which are normally supposed to exert anti-tumor functions [78]. However, in GBM, they play a central tumor-promoting role (reviewed in [79]). Microglia-derived enzymes, cytokines, and growth factors have been shown to directly lead to tumor proliferation and invasion, immunosuppression, and angiogenesis in primary brain tumors [80]. Microglia and macrophages can adopt distinct inflammatory types, namely the M1 and the M2 type. The M1 type is activated by type I cytokines (interferon (IFN) γ, tumor necrosis factor (TNF) α,) and lipopolysaccharide (LPS) and performs its antitumor immune function through pro-inflammatory cytokines and ROS generation. The M2 type, on the other hand, is reactivated by type II cytokines (interleukin (IL)-4, -10, -13) and promotes tumor growth and invasion by producing immunosuppressive (IL-10, transforming growth factor (TGF) β) and tumor survival factors (reviewed in [81]).

TSPO expression correlates with states of inflammation in the CNS [27] and is commonly used as a marker of inflammation in positron emission tomography (PET) studies [82]. TSPO expression is also increased in the surrounding microenvironment in GBM, including microglia and macrophages [66]. Various analyses revealed that TSPO ligands can modulate inflammatory and immune responses [83,84,85,86,87]. Therefore, a substantial body of evidence suggests that TSPO may have an immunomodulatory role in the CNS, but the precise mechanism remains unclear.

In principle, TSPO could be involved in the M1, anti-tumor/pro-inflammatory, or M2, pro-tumor/anti-inflammatory, response. A recent study in both a cellular and an animal model of inflammatory microglia demonstrated that elevated TSPO expression was restricted to M1 microglia [88]. These observations were confirmed by showing that TSPO expression was strongly associated with pro-, but not anti-inflammatory microglia, macrophages, and astrocytes, in vitro, and in vivo [89]. Contradictory to these results, TSPO expression was also shown to be consistently downregulated in macrophages activated to a pro-inflammatory M1 phenotype, whereas there was no difference in TSPO expression in M2 stimulated macrophages. These authors suggested that TSPO was involved in the negative regulation of inflammation, by promoting the M2 macrophage type [90]. Owen et al. demonstrated that stimulation with IFNγ and LPS caused an increased TSPO expression in rodent-derived microglia, but decreased TSPO expression in primary human microglia [91]. In addition, another study proposed that TSPO was directly involved in the modulation of the M1 and M2 phenotypes. TSPO silencing in human microglial C20 cells resulted in a more inflamed phenotype and increased the release of pro-inflammatory cytokines. In contrast, exposure of C20 cells to the TSPO ligand XBD173 attenuated the neuroinflammatory response [92]. Notably, *TSPO* mRNA expression was increased after stimulation with pro-inflammatory cytokines. These observations were accompanied by modulation of mitochondrial ROS and activation of the NF-κB signaling pathway which could then stimulate TSPO expression during the inflammatory response [92] and could link inflammation to another hallmark, namely cellular energetics.

Interestingly, the upregulation of various pro-inflammatory genes [93] and M2 macrophage and neutrophilic gene signatures are significantly associated with the mesenchymal phenotype [42], which has the highest percentage of microglia, macrophage, and lymphocyte infiltration [94]. Given that TSPO expression is also associated with the mesenchymal subtype [66], it would be interesting to see if TSPO is involved in this regulation.

Noteworthy, the M1/M2 polarization is a very simplified model, as microglia and macrophages undergo a spectrum of activation, which can differ, among other factors, with the type and length of a stimulus [95]. This also points to a more complex role of TSPO, which may depend on the context of the model. Therefore, the question to which extent TSPO might contribute to one phenotype or another and, accordingly, to the pro-inflammatory/anti-tumor or anti-inflammatory/pro-tumor responses cannot be answered yet.

### 2.2. Hallmarks of Cancer

#### 2.2.1. Sustaining Proliferative Signaling 

Normal tissue growth is tightly regulated through the release of growth-promoting and growth inhibitory signals [31]. Cancer cells, however, have developed mechanisms to proliferate independently of these regulatory signals. In GBM, alterations in RTK/Ras/PI3K enable the tumor to proliferate constantly [40]. For instance, overexpression of the epidermal growth factor receptor (EGFR) and inactivation of phosphatase and tensin homolog (PTEN) causes downstream activation of the growth- and survival-promoting PI3K/Akt/mTOR pathway [40,52,96,97,98].

Numerous studies support a pro-proliferative role of TSPO in GBM. High TSPO expression in the C6 rat glioma cell line is correlated to enhanced cell proliferation [19], and modulation of TSPO activity by the TSPO ligand PK11195 either had no effect or pro-proliferative effects in patient-derived glioma cell lines [99]. Likewise, overexpression of TSPO in C6 rat glioma cells enhanced proliferation as well as the ability to overcome contact-induced cell growth inhibition [100]. In addition, a more recent study revealed that the transfection of TSPO into Jurkat cells increased cell proliferation and motility [101] whereas lentiviral knockdown of TSPO reduced the proliferation rate in BV-2 mouse microglial cells [102].

In contrast, TSPO knockdown and TSPO ligands were able to promote proliferation and migration in glioblastoma U118MG cells due to a decrease in TSPO related apoptosis [103]. Knockout of TSPO with the CRISPR/Cas9 system in mouse GL261 glioma cells resulted in increased proliferation and viability in comparison to wild type cells [104]. This anti-proliferative role of TSPO is supported by pharmacological studies with a variety of TSPO ligands [22,105]. It is noteworthy that, reported earlier, the effects of TSPO ligands on cell proliferation are context-dependent and vary with ligand concentration, resulting in pro-proliferative effects at nanomolar concentrations and anti-proliferative effects at micromolar concentrations [19,106,107]. Context-dependent factors that could influence the role of TSPO are likely cell line-, species-, and signaling pathway-specific [102,108].

There is evidence that endogenous steroid hormones play a role in the development of gliomas. Epidemiological data suggest that female sex hormones play a tumor-suppressive role in GBM since the incidence rate of GBM is higher in men compared to women [109]. Previous studies have also shown that steroid hormone receptors such as ERβ as well as the testosterone-estradiol converting enzyme aromatase are expressed by some gliomas and glioblastomas [110,111,112]. Selective estrogen receptor modulators such as estradiol and 2-methoxyestradiol are shown to inhibit the proliferation of gliomas and induce cell death in experimental in vitro settings [109]. In line with these findings, an increased level of testosterone has been reported in patients with GBM [113]. In addition, androgen receptors are overexpressed in human GBM, and the genetic silencing of androgen receptors as well as their pharmacological inhibition, induce GBM cell death in vivo and in vitro [114,115,116]. Furthermore, the proliferation of GBM-derived cells was increased by testosterone, an effect that was antagonized by the androgen receptor antagonist flutamide [113]. The effects of the hormonal agonists and antagonists can either depend on classical steroid hormone receptor signaling or on alternative pathways [109]. In view of these findings, further studies are needed to elucidate the role of TSPO as a modulator of steroid synthesis in affecting the development and proliferation of glioma. Elucidating the role of hormonal pathways in gliomagenesis could eventually lead to the design of novel, preventive therapies.

The exact mechanism by which TSPO modulates cell proliferation is still unclear. Growing information indicates that TSPO impacts the bioenergetic profile of a cell by modulating ATP production, thus providing the energy for increased proliferation [18,25,101]. However, to gain a deeper understanding of the exact mechanisms and signaling pathways involved, further in-depth studies are required.

#### 2.2.2. Evading Growth Suppressors

Apart from sustaining proliferative signals, cancer cells also have the ability to escape growth inhibition and positively regulate cell proliferation through the loss of tumor suppressor genes such as *NF2*, *LKB1*, *RB*, and *TP53* [31]. The prevalence of mutations in the *RB* and *TP53* genes, though important drivers in many tumors, illustrates again that GBM is highly heterogeneous and depends on many distinct alterations: *RB* is mutated in only 6–11% of GBM cases and 27–33.8% of GBM bear mutations in the *TP53* gene [40,97]. Moreover, TP53-dependent cell cycle control can also be impaired by *MDM2* and *MDM4* amplification, which is the case in 12% and 4% of glioblastomas, respectively [117,118]. It is worth noting that the corresponding signaling pathways are nonetheless major targets of inactivating mutations in GBM and were altered in 78–79% of GBM and 87% of GBM cases for pRB and p53, respectively [40,97]. *RB* and *TP53* and the connected pathways play crucial roles in the inhibition of proliferation, predominantly by halting cells in the G1 phase. This delays entrance into the S phase, slowing down repair of DNA damage or ultimately causing apoptosis [52].

Changes in cell cycle regulation may enable cells to evade the control of growth suppressors. Early data suggested that TSPO is involved in the regulation of the cell cycle [119,120,121]. In a bioinformatical analysis investigating drug-response associated gene expression, *TSPO* has been found as a key driver gene for positive regulation of mitotic cell cycle phase transition [122]. Another publication revealed *TSPO* as a critical, differentially expressed gene in neuroblastoma with cyclin-dependent kinase (CDK) 2 silencing. *TSPO* was identified as a key target gene of CDK2, and CDK2 may be involved in tumor progression via the regulation of the interaction of TSPO and CDK1 [123]. Other publications corroborated an interaction of TSPO with cell cycle-related genes, e.g., in the U118MG glioblastoma cell line. The same authors showed that the down-regulation of TSPO expression caused an increase of cells in the S and G2/M phase and a decrease of cells in the G1/G0 phase [124]. An independent group obtained similar results with an enhanced ratio of TSPO knockout cells in the S phase [104].

Results with pharmacological inhibitors of TSPO also support evidence that TSPO modulates cell cycle progression. For instance, TSPO ligands inhibit cell proliferation by halting these cells in the G1/G0 phase and therefore inhibiting the progression to the S and G2/M phase [125,126]. Short-term treatment with PK11195 resulted in a reduction of the S and G2/M phase and a consequent increase of the G1/0 phase, whereas longer treatment periods caused a decrease of cells in the S phase and accumulation in the G2/M phase [124]. A follow-up study confirmed that exposure of U118MG glioblastoma cells to PK11195 induced time-dependent changes in the regulation of the cell cycle and cell proliferation. These functional effects were most likely achieved by modulating the expression of immediate early genes and cell cycle regulators. These results suggest that TSPO exerts such effects as a part of the mitochondrial-to-nucleus signaling pathway that modulates nuclear gene expression [127].

In summary, the latest evidence hints at a role for TSPO in cell cycle regulation. In light of alterations of various important cell cycle regulators and signaling pathways in GBM, it will be interesting to understand the interactions of TSPO with cell cycle checkpoint molecules and tumor suppressors in more detail.

#### 2.2.3. Resisting Cell Death

Cell death plays an important role in suppressing cancer development and deregulation of cell death mechanisms—such as apoptosis, autophagy, and necrosis—is one of the main reasons for GBM treatment failure [128,129,130]. Apoptosis can be divided into an extrinsic (mediated by death receptors) and an intrinsic (mediated by mitochondria) arm, which both lead to the activation of the executioner caspases 3 and 7 [131]. Thereby, mitochondria play a key role in cell death signaling by initiating the caspase cascade through outer mitochondrial membrane permeabilization and subsequent release of cytochrome c [132]. It is, therefore, reasonable to suggest that TSPO, as a mitochondrial protein, has one of its major functions here. Knockdown studies conducted over the last decade revealed that downregulation of TSPO reduced the apoptotic rate, implying a direct [133,134] or indirect pro-apoptotic role of TSPO, for example by reducing the pro-apoptotic effect of glutamate [135].

An apoptosis-promoting role of TSPO is also supported by pharmacological evidence. For instance, TSPO ligands were able to induce cell death in colorectal cancer cell lines [120], in chronic lymphocytic leukemia cells [136], as well as in neuroblastoma cell lines in a dose-dependent manner [126]. In addition, the exposure of several glioma and GBM cell lines to various TSPO ligands resulted in the collapse of the mitochondrial membrane potential (ΔΨm), activation of the caspase cascade, and subsequent apoptosis [137,138,139].

In contrast, the TSPO ligands PK11195 and Ro5 4864 were also described to reduce apoptosis in different glioma cell lines, as well as in human monocytic cells and in a rat model of myocardial ischemia-reperfusion [19,22,140,141]. This points to a notorious problem with TSPO ligands, namely their, often not well-defined, function as agonists or antagonists as well as possible concentration-dependent effects. Results from TSPO ligand assays should always be carefully considered, as even the most advanced synthetic ligands tend to yield off target-effects, implying that a pro-apoptotic effect of higher ligand concentrations may rather depend on interactions with other targets [107,108,142,143,144,145].

The mechanism by which TSPO regulates apoptosis is still an enigma. In 1995, it was suggested that TSPO, together with the voltage-dependent anion channel 1 (VDAC1) and the adenine nucleotide transporter (ANT), form the mitochondrial permeability transition pore (mPTP) [146]. Moreover, a variety of studies reported that both endogenous and synthetic TSPO ligands modulate the activity of the mPTP [147,148,149]. Since TSPO is proposed as a critical regulator of this complex through modulation of VDAC1 conductance [150], regulation of cell death may indeed be the most important function of TSPO.

The effects of TSPO include the regulation of redox stress homeostasis [21,151] and ΔΨm [134,152], which can eventually lead to apoptosis by inducing cytochrome c release, caspase activation, and DNA fragmentation [19,21,22,133]. Recently, however, the role of TSPO on mPTP function has been challenged. For instance, a study using conditional liver- and heart-specific TSPO^−/−^ mice revealed that TSPO does not function as a member of the mPTP. Furthermore, TSPO ligands had no impact on mPTP activity and the outer mitochondria membrane regulation of mPTP activity occurred through mechanisms independent of TSPO [143]. In a study that used the CRISPR/Cas9 system, TSPO knockout MA-10 cells displayed a significantly reduced ΔΨm compared to control, as well as resistance to apoptosis [153]. Another study investigating the role of TSPO in ischemia/reperfusion injury revealed that the upregulation of ROS and oxidative stress, as well as the collapse of the ΔΨm, mPTP opening, and apoptosis induced through anoxia/reoxygenation, were completely abolished by TSPO knockdown [154]. Whether TSPO modulates the ΔΨm through the mPTP, therefore, remains controversial. Based on gene expression analysis data, it was proposed that TSPO might regulate the expression of genes associated with apoptotic processes through mechanisms such as ΔΨm collapse, ROS generation, Ca^2+^ release, and ATP production, which, as a functional consequence, could potentially lead to cell death [127]. Further, the described mechanism may well depend on the respective context in view of tissue of origin, microenvironment, and experimental conditions.

In summary, mitochondria are tightly linked to cell death through a variety of mechanisms. Considering TSPOs position in the mitochondrial membrane and according to experimental evidence, published data clearly indicate that TSPO is involved in the regulation of cell death. However, it remains open in what exact mechanistic way and in which context TSPO modulates the resistance of GBM cells to apoptotic stimuli and how this is related to other cell death mechanisms such as autophagy and necrosis.

#### 2.2.4. Enabling Replicative Immortality

With each round of cellular replication, telomeres shorten until they finally reach a critical length that is unable to support the stable formation of shelterin protein complexes that protect telomeres from DNA damage surveillance mechanisms [155]. In order to divide uncontrollably, cancer cells need to acquire an infinite capacity to replicate. To meet these conditions, they alter the expression of genes such as *TERT* which encodes the telomerase reverse transcriptase [156]. By extending the length of the telomeres, this enzyme actively contributes to the capacity of unlimited proliferation. *TERT* mutations in GBM occur frequently with ~83% of GBM *IDH* wildtype being *TERT* mutated [157]. On the other hand, GBM *IDH* mutated tumors have been reported to have a lower incidence of *TERT* mutation [158], which makes *TERT* status a valuable additive tool for defining GBM prognosis.

Even though TSPO might modulate the aging of cells through indirect mechanisms such as controlling the energy supply, cell death, and angiogenesis, our literature research revealed no data that could link TSPO to immortality-related mechanisms at this time.

#### 2.2.5. Inducing Angiogenesis

Alterations in angiogenic pathways contribute largely to the aggressiveness of GBM. GBM exhibits an extensive network of abnormal vasculature to supply itself with nutrients and oxygen, and several upregulated angiogenic receptors and factors stimulate angiogenesis signaling pathways (reviewed in [159]). Neo-vascularization in GBM is mainly mediated by vascular endothelial growth factor (VEGF), basic fibroblast growth factors (bFGF), hepatocyte growth factor (HGF), platelet-derived growth factor (PDGF), transforming growth factor-β (TGF-β), matrix metallopeptidases (MMPs), and angiopoietins (Angs) (reviewed by [160]). Unfortunately, the survival benefit of treatment with angiogenesis inhibitors is limited, since tumor cells can evade them through the modulation of evasive resistance pathways (reviewed in [161]).

It has been suggested that TSPO may be involved in the angiogenesis of tumors [162]. For instance, tumors developed from U118MG TSPO knockdown cells exhibited expanded angiogenesis in chorioallantois membranes of chicken embryos compared to tumors developed from scrambled controls [103]. In a TSPO knockout GL261 xenograft glioma model, extensive hemorrhages were observed. Moreover, the levels of angiogenesis regulators such as HIF-1α, VEGF-A, MMP2, and IL-8 were significantly increased in TSPO knockout gliomas in comparison to wild type. The authors concluded that TSPO-deficiency triggers HIF-1α upregulation, leading to a subsequent increase in key angiogenesis regulators that fueled angiogenesis and a tumor-promoting microenvironment [104]. Finally, a role for TSPO in the aberrant proliferation and migration of vascular smooth muscle cells (VSMCs) was demonstrated [18]. Briefly, overexpressing TSPO in VSMC cells had a positive effect on proliferation and migration, whereas knockdown of TSPO or modulating TSPO function with its ligands PK11195 and Ro5 4864 resulted in a significant decrease in proliferation and migration of PDGF-BB treated VSMCs [18].

To date, only a few mechanistic studies on TSPO and angiogenesis are available and further research is needed to understand the role of TSPO in the angiogenesis of GBM. Available results suggest that high levels of TSPO counteract angiogenesis in GBM potentially through modulation of genes connected with the canonical pathway for angiogenesis [127].

#### 2.2.6. Activating Invasion and Metastasis

One of the major reasons for GBM recurrence is the migration and diffuse invasion of tumor cells into the surrounding brain. The background mechanism that regulates migration and invasion is an epithelial-to-mesenchymal transition (EMT) [163,164]. Through activation of this program, tumor cells develop the ability to detach from their primary site and invade surrounding tissue, hence, acquire an aggressive, invasive phenotype [165]. In the context of EMT, changes in the shape of tumor cells, as well as aberrations in the attachment of cancer cells to other cells and to the extracellular matrix (ECM), are highly relevant [31,166,167].

High expression of TSPO is associated with invasiveness in several cancers including GBM [168]. In the C6 rat glioma cell line, the overexpression of TSPO increased the ability to overcome contact-dependent inhibition of cell growth. This was accompanied by an increase in the motility rate and the transmigrative phenotype of C6 cells [100]. Wu and Gallo (2013) demonstrated, by means of transient overexpression or silencing of TSPO, that TSPO contributes to the migration of breast cancer cells [23]. In particular, TSPO overexpression in a poorly migratory breast cancer cell line resulted in increased migration, whereas the silencing of TSPO in a highly invasive breast cancer cell line decreased migratory capabilities [23]. In contrast, knockdown of TSPO in U118MG resulted in decreased adhesion to extracellular matrix proteins such as collagen I and IV, fibronectin, laminin I, and fibrinogen as well as in an increase in migratory capability [103]. In addition, treatment of U118MG with PK11195 resulted in an upregulation of genes related to migration, which was confirmed by microscopic observations showing the congregation and segregation of cells [127].

Adhesion molecules are involved in cell-cell interactions and contact to the ECM, and are therefore important actors in migration and invasion. Early studies have observed that vascular cell adhesion molecule-1 (VCAM-1) is aberrantly expressed in several cancers, including GBM [169,170]. Interestingly, studies in vascular endothelial cells revealed that TSPO modulates VCAM-1 and ICAM-1 expression. Overexpression of TSPO inhibited TNFα-induced, as well as phorbol 12-myristate 13-acetate (PMA)-induced, VCAM-1 and ICAM-1 expression in a dose-dependent manner [171].

In view of these rather scarce results, the underlying mechanisms whereby TSPO influences cell migration are unknown at this time. To our knowledge, a link between TSPO, VCAM-1, and the infiltrative phenotype of GBM has not been established yet. However, it is conceivable that TSPO regulates migration and invasion through modulating the expression of adhesion molecules and/or affects the cellular energy production necessary for migration [23,127]. Identification of the underlying molecular mechanisms may provide a deeper understanding.

### 2.3. Emerging Hallmarks

#### 2.3.1. Evading Immune Destruction

Cancer cells develop mechanisms to overcome immune surveillance and thus evade anti-tumor immune response [31]. GBM is able to avoid immune destruction through a variety of mechanisms including the creation of an immunosuppressive microenvironment, decreased presentation of antigens, exploiting immune checkpoints, and recruiting of tumor-associated macrophages (TAM) and microglia (reviewed in [172]).

Although microglia are considered the main immune cells of the CNS, TSPO is also able to modulate the activity of other immune populations. A recent study revealed that the TSPO ligands FGIN1-27 and PK11195 inhibited cytokine production of CD4^+^ T-cells in a dose- and time-dependent manner [83]. Furthermore, the specific TSPO ligand vinopectine was shown to decrease the expression of several important regulators for T- and B-cell responses in plasmacytoid dendritic cells (pDCs) [173]. In addition, vinopectine significantly inhibits the Toll-like receptor 9 (TLR9) signaling pathway and reduces the secretion of inflammatory cytokines. The silencing of TSPO expression abrogated this inhibition [173]. These results are important, as DCs connect the innate and adaptive immunity and can present antigens. As such, they have the ability to promote anti-tumor T-cell responses, and can enhance tumor immunogenicity [174]. Although they are not generally present in the CNS, they can be recruited in response to pathological stimuli [175]. Interestingly, in glioma, the TLR9 expression by pDCS is downregulated and both TLR9 and the manipulation of DC activity are proposed to be attractive targets in GBM immunotherapy [174,175,176,177].

It has also been shown that the upregulation of TSPO by chemical TSPO ligands can modulate the inflammatory response through secretion of a variety of cytokines including TNFα, IL-1, and IL-6 [84,178,179,180]. TSPO expression can be triggered by TNFα as a response to the secondary induction of the pro-inflammatory cytokine IL-8. On the other hand, TSPO activation by its natural ligands blocks the production of IL-8 which controls ROS levels and stabilizes mitochondrial membrane integrity [181]. These studies point out that TSPO mediates the production of ROS which could then lead to the induction of inflammatory cytokines such as IL-1, IL-6, and TNFα. These cytokines are reported to exert differential effects on effector T-cell activity and may therefore play a critical role in the regulation of tumor-immune rejection in GBM [182,183].

Interestingly, the exposure of U118MG glioblastoma cells to PK11195 downregulates the expression of genes involved in immunomodulation [127]. Moreover, key immune response regulators control TSPO expression itself. For instance, ROS is proposed to activate protein kinase Cε (PKCε), which signals through the mitogen-activated protein kinase (MAPK) pathway and modulates TSPO expression through c-Jun and STAT3 transcription factors [63], for detailed reviews, see [184,185], which all together modulate the immune response via complex interconnections [186,187,188].

On a final note, it must be taken into account that the tumor microenvironment is composed of various cell populations that communicate through a complex dynamic network of cytokines, chemokines, and growth factors (reviewed in [189]). Thus, understanding which interactions take place and how they are influenced by TSPO will be a challenge in future studies.

#### 2.3.2. Reprogramming Energy Metabolism

Cancer cells can adapt their energy metabolism into several directions to maintain their chronic, often uncontrolled cell proliferation. For example, increased aerobic glycolysis (Warburg effect) contributes to the malignant progression of GBM, enabling tumor expansion and providing apoptotic resistance through a high rate of glucose uptake, lactate production, and acidification of the tumor environment (reviewed in [190,191,192,193]). Furthermore, GBM cells also use intermediates of the tricarboxylic acid cycle and oxidative phosphorylation to generate energy (reviewed in [194]).

A growing amount of evidence supports the role of TSPO in cellular energy production. For instance, modulating TSPO expression by selective ligands or genetic editing resulted in altered mitochondrial energy metabolism [195,196]. Microglia isolated from TSPO knockout mice displayed an altered oxygen consumption as well as a significantly reduced ATP production compared to controls [197]. Stable overexpression of TSPO in Jurkat cells with low endogenous TSPO expression caused an upregulation of genes involved in the mitochondrial electron transport chain for energy production. Furthermore, mitochondrial ATP production, as well as excitability of these cells, was increased, resulting in increased proliferation and motility as functional consequences [101]. In line with these findings, TSPO knockout in C20 microglia correlated with decreased mitochondrial membrane potential, cytosolic Ca^2+^ levels as well as a reduced respiratory function [25].

Additionally, TSPO appears to play a key role in glioma growth and malignancy by controlling the metabolic balance between oxidative phosphorylation and glycolysis. TSPO knockout in a glioma animal model as well as in patient-derived cells resulted in increased mitochondrial fragmentation, reduced ATP production, and decreased mitochondrial oxidative phosphorylation. Moreover, TSPO knockout caused a metabolic shift towards glycolysis, evidenced by increased glucose uptake and lactic acid conversion [104]. However, also contradictory results have been published. For example, a study investigating the role of TSPO in mPTP formation and OMM regulation demonstrated that there was no difference in the basal oxygen consumption rate between liver-specific TSPO^-/-^ mice and controls. Moreover, mitochondria lacking TSPO displayed the same rates of ADP- and uncoupler-stimulated respiration as control mitochondria [143].

Strong evidence suggests that TSPO modulates ROS generation and consequently the mitochondrial respiratory chain [21,24,198,199], providing a possible link between TSPO and the F_O_F_1_-ATPase. In 2009, it was shown that TSPO ligands could modulate energy production in mitochondria by controlling phosphorylation of the F_O_F_1_-ATPase subunit c [200]. Veenman et al. revealed that inhibition of the F_O_ subunit by oligomycin prevented TSPO ligand-induced apoptosis in glioblastoma cells [201]. This link is further supported by the strong interaction of TSPO with VDAC1. This outer mitochondrial membrane protein is described to control cellular energy and metabolic homeostasis [202,203]. Interestingly, depletion of VDAC1 in GBM cancer xenografts altered the expression of key proteins related to glycolysis, the tricarboxylic acid cycle, and oxidative phosphorylation including the expression level of the ATPase [204]. In addition, the downregulation of VDAC1 caused decreased TSPO expression as well as translocation of TSPO to the nucleus [204]. Conversely, TSPO knockout in C20 microglia resulted in reduced levels of VDAC1 expression [25]. The close association of these two important metabolic regulators may modulate ATPase activity and other metabolic key proteins to adjust the energy status of a cell under various conditions including the high energy demand of proliferating cancer cells. Notably, Liu et al. proposed that TSPO may modulate the ATPase via a direct interaction between the ATP “synthasome” complex (composed of ATPase, phosphate carrier, and ANT) and the PBR complex composed of TSPO, VDAC1, and ANT [101].

In summary, the majority of these findings indicate that TSPO, probably in interaction with other mitochondrial proteins, contributes to the adapted metabolism of GBM by modulating the expression of genes involved in key metabolic functions. However, considering the high expression of TSPO in GBM and its tendency to fuel aerobic glycolysis, it is difficult to understand why TSPO deficiency shifts the metabolic balance to a more glycolytic phenotype by increasing the expression of key glycolytic genes [104]. These findings point to a complex role of TSPO in tumor metabolism, which is not fully understood at this time. Of note, many functions of TSPO may directly depend on energy metabolism, influencing the diversity of malignant hallmarks in GBM. An overview of TSPO´s effects on different glioma cell lines is given in Table 1.

## 3. In Vivo-Monitoring of TSPO

Early on, the strong potential of TSPO as a non-invasive marker in glioma was recognized, based on its overexpression in tumors compared to the normal brain. This observation is not only relevant in a diagnostic context, but also for a possible future therapeutic context, where the response to TSPO-modulating agents could be directly monitored in true time.

Pre-clinical studies using TSPO PET ligands for in vivo imaging of gliomas revealed promising results [207,208], which were corroborated in humans by first-generation PET-tracers such as ^11^C-PK11195 [209,210]. These, however, were rather impractical for human PET due to the high unspecific binding and the short half-life, which restricts the use to centers with on-site cyclotron only [211,212]. These disadvantages pushed the evolution of new TSPO ligands with better tracer characteristics, and several new high-affinity and selective TSPO ligands like ^18^F-DPA-714 or ^18^F-GE-180 were developed. They are characterized by a longer half-life due to the radiolabeling with ^18^F, show high tumor-to-brain contrast and their uptake positively correlates with WHO grades, suggesting a promising tool for GBM diagnostic imaging [211,213].

Interestingly, TSPO-PET not only visualizes the tumor mass but also activated microglia in TAM and potentially additional inflamed components of GBM [66,214]. In view of its suspected role in the immune escape, TSPO-PET could therefore also be a promising way to visualize the reactive microenvironment.

Currently, however, imaging of gliomas with PET TSPO ligands is not standardized, and it remains unclear to which degree the TSPO PET signal reflects tumor cells or activated microglia. Furthermore, the influence of a common polymorphism (rs6971) in the TSPO gene on the binding affinity and consecutively on the PET signal is still under investigation [215]. Therefore, additional clinical studies are needed to develop these radiotracers for the standard evaluation of GBM.

## 4. Conclusions

**Figure 2:**
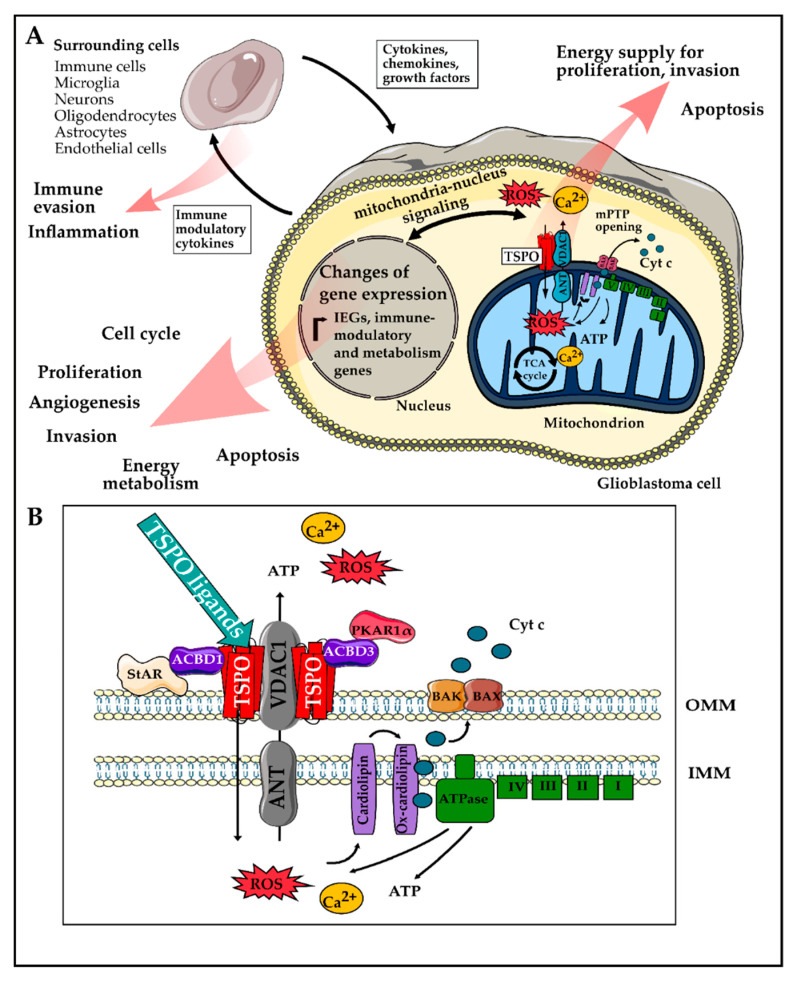
Overview of the mechanisms of how TSPO could modulate the hallmarks of Glioblastoma (GBM). (**A**) TSPO, together with other mitochondrial proteins such as voltage-dependent anion channel (VDAC), adenine nucleotide transporter (ANT), and ATPase can modulate mitochondrial Ca^2+^ release, ATP production, and reactive oxygen species (ROS) generation. The latter can then lead to the release of cyt c, which triggers the mitochondrial apoptosis cascade and ultimately apoptosis. An increase in ATP production, on the other hand, could provide energy for enhanced proliferation and invasion of GBM cells. The mitochondrial ROS, ATP, and Ca^2+^ release are also considered as a part of the mitochondria to nucleus signaling, which can modulate the expression of immediate early genes and transcription factors, as well as metabolism-related and immune-modulatory genes [124,127]. Several hallmarks of GBM can be modulated as a functional consequence of these gene expression changes. Furthermore, the immune-modulatory factors and cytokines secreted by the tumor cell can modulate surrounding cells contributing to immune escape and a tumor-promoting microenvironment [182,183]. (**B**) Close up showing the proposed working mechanism: TSPO is located in the outer mitochondrial membrane and can be found in close proximity to several cytosolic proteins such as StAR, ACBD1, ACBD3, and PKAR1α, which have been described to play a role in steroidogenesis (reviewed by [216]). Furthermore, binding of TSPO ligands to TSPO, in interaction with VDAC1, can modulate ROS and ATP production by modifying the activity of the ATPase [200,201]. An increase in the levels of ROS can result in cardiolipin oxidation and opening of the mPTP, consisting of VDAC1 and ANT [21,75,151]. The opening of the mPTP causes the release of ATP, ROS, and Ca^2+^ from the mitochondria into the cytosol and the collapse of the ΔΨm. The depolarization then leads to the opening of BAK/BAX channels, allowing the passage of cyt c into the cytosol. Abbreviations: ACBD, acyl-CoA-binding domain protein; cyt c, cytochrome c; IEG, immediate early genes; ROS, reactive oxygen species; mPTP, mitochondrial permeability pore; ΔΨm, mitochondrial membrane potential; PRKAR1α, protein kinase cAMP-dependent type I regulatory subunit alpha; StAR, steroidogenic acute regulatory protein.

The highly proliferative, invasive, and immunosuppressive nature of GBM contributes to poor clinical outcomes and makes GBM significantly challenging to treat. Essentially all hallmarks of cancer, as proposed by Hanahan and Weinberg [31], are reflected in the pathophysiology of GBM. In this review, we summarized experimental evidence suggesting that TSPO might play a role in most of these hallmarks and may be a central modulator of malignancy in GBM. In particular, TSPO’s ability to modulate gene expression and cellular energetics could explain these effects, however, the exact mechanisms remain unclear at this time in most cases. Figure 2 shows a graphical summary of possible mechanisms by which TSPO could influence cancer hallmarks as well as an overview of the most important TSPO-interacting proteins.

Of note, TSPO ligands are often-used investigational tools. However, it must be taken into consideration that these ligands may have off-target effects [102], which makes further validation with genetic tools essential. The role of TSPO in resisting apoptosis, reprogramming cellular energetics, and evading immune destruction might hold promise for future therapeutic interventions. However, targeting TSPO is not ready for clinical use at this time. Since GBM can effectively escape radio- and chemotherapy through a flexible adjustment of metabolism, cell death mechanisms, and the immune system, effective treatments must eliminate these escape routes. Modulation of TSPO activity through specific TSPO ligands could pave the way for attenuating this flexibility and making the tumor more susceptible to tumor-specific treatments. The development of more specific TSPO ligands could also represent a promising pipeline to monitor GBM and its microenvironment during diagnosis and treatment.

## Figures and Tables

**Table 1:** Overview of experimental data listing the effects of TSPO modification on different glioma cell lines.

Treatment, Ligand	Cell Line	Effect on GBM	Year	Reference
TSPO KO	Mouse GL261 cells Human GBM1B cells	↑ glioma growth and angiogenesis in vivo, ↑ fragmented mitochondria, glucose uptake, lactic acid conversion, ↑ ROS, ↑ glycolysis, ↓ oxidative phosphorylation and ATP production	2020	[104]
PK11195 (25 µM)	Human U118MG cells	Changes in expression of immediate early genes and transcription factors, functional changes related to cell cycle, cell death, proliferation, migration, cell viability, inflammatory, immune response, and tumorigenesis	2017	[127]
PK11195 (25 µM), TSPO KD	Human U118MG cells	Changes in gene expression related to cell cycle, apoptosis, oxidative stress, immune response, DNA repair, adhesion	2014	[124]
Ammonium chloride + PK11196, Ro5 4864, FGN-1-27 (1 nM–100 µM)	Human U118MG cells	↓ cell death at nanomolar concentration, ↑ mitochondrial dysfunction and cell death at micromolar concentration	2014	[142]
Quinazoline derivate compound 19 (10 nM–100 µM)	Human U343 cells	↓ proliferation, dose-dependent, ↑ dissipation of ΔΨm	2014	[138]
irDE-MPIGA (1.25 × 10^−3^ nmol/L + 10^6^ cells), PIGA (2.5 µM) irDE-MPIGA + TSPO KD	Human U87MG cells	↓ cell viability without cell cycle arrest, ↑ ΔΨm dissipation, No effect on ATPase activity No reduction of cell viability	2014	[205]
PK11195 (25 µM), TSPO KD	Human U118MG cells	↑ tumor growth, ↑ angiogenesis, ↑ migration, ↓ adhesion of ECM	2012	[103]
Sodium nitroprusside + PK11195 (25 µM), TSPO KD	Human U118MG cells	↓ cell death and collapse of ΔΨm, restoration of metabolic activity	2012	[151]
TSPO KD Glutamate + TSPO KD	Human U118MG cells, Rat C6 cells	Changes in glutamate metabolism ↓ DNA fragmentation	2012	[135]
PPIX (1–30 µM) + light + TSPO KD GSH + PPIX	Human U118MG cells	↑ cell death, ↑ PPIX accumulation in cell and mitochondria ↑ PPIX accumulation in mitochondria	2012	[26]
Oxazolacetamide compound 6d (30 times of K_i_ value)	Human U87MG cells	↑ cell proliferation/viability, ↑ dissipation of ΔΨm,	2011	[105]
CoCl_2_ + PK11195 (25 µM), TSPO KD	Human U118MG cells	↓ cell death, ↓ ΔΨm collapse, ↓ ROS generation, ↓ cardiolipin oxidation	2009	[21]
ErPC3 + PK 11195, Ro5 4864 (25 µM–100 µM)	Human U87MG cells Human A172 cells Human U118MG cells	↓ ErPC3 induced apoptosis, ↓ cytochrome c release, processing of caspase 9 and 3	2008	[22]
TSPO overexpression PK11195 (1–100 µM) + TSPO overexpression	Rat C6 cells	↑ proliferation, ↑ migration and transmigrative capabilities, ↑ anti-proliferative effect	2007	[100]
Ro5 4864 (10 nM)	Rat C6 cells, human T89G cells	↓ cell death	2004	[19]
PK11195, Ro5 4864 (10 nM)	Rat C6 cells, human T98G cells	↑ mitochondrial replication, shift of mitochondria from peripheral cytoplasm to the perinuclear region	1991	[206]

Abbreviations: ΔΨm, mitochondrial membrane potential; ECM, extracellular matrix; ErPC3, erucyl-phospho-homocholine; CoCl_2,_ cobalt chloride; GSH, glutathione; KD, knockdown; KO, knockout; PPIX, protoporphyrin IX; ROS, reactive oxygen species.
